# Management of burn patients undergoing surgery while on extracorporeal membrane oxygenation (ECMO): clinical experience and a standardized perioperative protocol

**DOI:** 10.1186/s13741-025-00572-2

**Published:** 2025-08-20

**Authors:** Sonja Verena Schmidt, Elisabete Macedo Santos, Marius Drysch, Flemming Puscz, Felix Reinkemeier, Dirk Buchwald, Peter K. Zahn, Marcus Lehnhardt, Jannik Hinzmann, Christoph Wallner

**Affiliations:** 1 Department of Plastic Surgery, BG University Hospital Bergmannsheil, Ruhr University Bochum, 44789 Bochum, Germany; 2 Department of Anesthesiology, BG University Hospital Bergmannsheil, Ruhr University Bochum, 44789 Bochum, Germany; 3 Department of Cardiothoracic Surgery, BG University Hospital Bergmannsheil, Ruhr University Bochum, 44789 Bochum, Germany

**Keywords:** ECMO, Burn surgery, Anticoagulation, Perioperative medicine, Intensive care

## Abstract

Severe burn injuries complicated by acute respiratory failure present unique challenges in critical care medicine. Although the use of veno-venous extracorporeal membrane oxygenation (V-V ECMO) can offer life-saving support for this patient cohort, the perioperative management of burn patients on ECMO remains poorly standardized, and evidence-based guidelines are lacking. This perspective outlines the experiences gained from managing burn patients undergoing surgery while on V-V ECMO at a major burn center. Over a 3-year period, 14 patients with an average burned total body surface area (TBSA) involvement of 41% were treated with ECMO support. Several key strategies contributed to the safe surgical management of these patients. Looking ahead, there is a clear need for multicenter registry data and collaborative efforts to establish standardized perioperative protocols for burn patients receiving ECMO. Individualized anticoagulation management using point-of-care techniques such as thromboelastography, and the evaluation of optimal surgical timing strategies, will be essential areas for future research. In conclusion, interdisciplinary teamwork and structured perioperative management protocols can enable safe surgical treatment of burn patients on ECMO. Broader collaboration and standardized data collection are crucial steps to improving outcomes and establishing best practices for this complex patient population.

## Background

In burn patients, acute respiratory distress syndrome (ARDS) and respiratory failure are serious complications that carry a high mortality risk. ARDS in this population can result from inhalation injury, excessive fluid resuscitation, ventilator-associated pneumonia, or pre-existing pulmonary disease. Initial management relies on supportive strategies such as lung-protective ventilation and prone positioning. However, veno-venous extracorporeal membrane oxygenation (V-V ECMO) may be required as a rescue therapy in cases of refractory respiratory failure.

The most common reason for V-V ECMO in burn patients is severe ARDS that does not respond to the maximum level of conventional therapy, which is usually triggered by inhalation trauma or secondary infections such as pneumonia. Other indications include life-threatening hypoxemia (PaO_2_/FiO_2_ratio < 80 mmHg despite optimal care), refractory hypercapnia with acidosis, and poor respiratory mechanics that are incompatible with protective ventilation strategies (Butchtele and Levy 2024; Chiu et al. 2022). However, the decision to initiate ECMO in burn patients requires especially cautious consideration (Fina et al. 2020). Due to the unique pathophysiology of this group, which is characterized by extensive tissue injury, a high risk of infection, and bleeding susceptibility, the selection criteria tend to be more restrictive than for other ARDS populations. Current consensus guidelines, such as those from the Extracorporeal Life Support Organization (ELSO), emphasize the importance of individualized, multidisciplinary decision-making with a focus on early identification, precise timing, and rigorous hemostatic management to optimize benefits for this high-risk group.

Compared to other patient cohorts, severely burned patients are a particularly challenging group because they often sustain extensive injuries. During the period of ECMO treatment, surgery may be unavoidable as debridement of necrosis or coverage of wounds is essential in order to prevent serious infections. In these cases, careful preparation and planning are crucial. One of the most challenging aspects in this context is anticoagulation. Due to the simultaneous risk of thrombosis within the ECMO system and bleeding from the burn injuries or the donor sites, the anticoagulation strategy must be carefully balanced. Most centers use unfractionated heparin for therapeutic anticoagulation, yet there is wide variability in dosing, monitoring, and perioperative adjustments (Frost et al. 2021). Furthermore, less than half of burn centers performing ECMO therapy have a standardized protocol (Frost et al. 2021). For monitoring heparin administration, activated partial thromboplastin time (aPTT) and anti-factor Xa activity are commonly used to guide therapy, but their correlation also has limitations and does not cover all aspects of coagulation ( 2017; Hebert et al. 2022). In recent years, ECMO programs are shifting toward more individualized and even low- or no-anticoagulation protocols in selected patient populations, particularly those at high risk for bleeding (Jung et al. 2025). Up to 50% of patients on ECMO experience bleeding complications, with approximately 7.7% developing life-threatening hemorrhages. Elevated aPTT levels have been associated with an increased risk of bleeding events (Martucci et al. 2024). Especially burn patients represent a unique cohort, sometimes requiring multiple surgeries, and therefore demand an individualized and thoughtful approach to anticoagulation and perioperative management to reduce the risk for life-threatening events of bleeding.

Over the past few years, we have managed several cases of severely burned patients requiring V-V ECMO, in some of whom surgical intervention was unavoidable despite the complexity of their critical condition. Due to the challenges of balancing surgical timing, anticoagulation management, and ECMO support, we were faced with different perioperative strategies and encountered a wide range of clinical scenarios. Through these experiences, we were able to identify key factors that influence outcomes and gradually developed a standardized perioperative approach. Based on this experience, we propose a structured protocol to guide the surgical management of burn patients on ECMO. Subsequently, we present an overview of our recent patient cohort and associated complications that served as the foundation for the development of the protocol.

## Institutional experience

Between 2022 and 2024, a total of 14 burn patients (11 male and 3 female) with an average total body surface area (TBSA) burn injury of 41% were treated with veno-venous extracorporeal membrane oxygenation (V-V ECMO) in our specialist burn unit. The mean duration of ECMO support was 12 days (range 1–29), the shortest of which was in a patient who died on the day ECMO was initiated. On average, 11.82% TBSA was excised during the ECMO period. Skin graft take was satisfactory in all cases. In some patients, a further grafting procedure was performed as part of a staged wound coverage strategy, for example, following the application of a dermal substitute, or due to reduced physiological reserves, rather than as a result of primary graft failure.

The mean number of red blood cell (RBC) units transfused per patient during the time of ECMO was 14.92. Bleeding complications were observed in 7 patients; however, only one case (hemothorax) required surgical intervention. All other bleeding episodes were managed conservatively through heparin adjustment and local measures without any hemodynamically significant events. No thromboembolic complications occurred. Only in 2 procedures did we decide to end the operation early due to intraoperative hemodynamic instability. Oxygenator exchange was required in 2 patients (14%) and was performed electively. Thanks to the standardized protocol and close interdisciplinary coordination, unforeseen intraoperative complications were largely avoided. The observed mortality rate of 64% (9 of 14 patients) reflects the underlying critical condition of this cohort rather than surgical or ECMO-related adverse events.

While most patients were otherwise healthy, relevant comorbidities in the cohort included arterial hypertension (four cases), chronic obstructive pulmonary disease (three cases), asthma (two cases), obesity (two cases), and psychiatric or substance-related disorders (four cases). These were considered in perioperative planning, but did not preclude surgical or ECMO management.

All descriptive values (means, percentages) were calculated using Microsoft Excel. Due to the small sample size and descriptive nature of the report, no formal statistical testing was performed (Table 1).

**Table 1:** Overview of burn patients with V-V ECMO support between 2022 and 2024

Number of patients treated with V-V ECMO	14
Male patients	79%
Average age in years	43.14
Average burned TBSA	41%
Average number of surgeries during ECMO	0.86
Mean % of TBSA excised during ECMO	11.82%
Average number of RBC units transfused during ECMO per patient	14.92
Average days of ECMO therapy	12 days
Change of oxygenator due to clotting	14%
Deceased	64%

## Establishment of standardized protocol

Preoperative planning:

- Heparin is administered following a low-dose anticoagulation protocol, aiming for a target activated partial thromboplastin time (aPTT) of 40 s after an initial bolus of 5000 IU of heparin during ECMO implantation
- Crossmatch sufficient amount red blood cell units (RBCs), at least 1 RBCs for 8% wound area (donor site included)
- Make a differentiated plan of which areas to excise and which areas to graft (take bleeding of donor sites into consideration)
- Consider epifascial debridement instead of tangential excision if large areas (> 10% TBSA) need to be excised
- Establishment of system for advanced hemodynamic monitoring, such as pulse contour cardiac output monitoring (for static values and trends)

Four hours before surgery:

- Determination of hemoglobin levels and substitution of RBCs to reach a hemoglobin of at least 8 mg/dl
- Determination of calcium levels (co-factor for coagulation) and substitution until physiological levels are reached (2.09–2.54 mmol/l)
- Determination of factor XIII (fibrin-stabilizing factor) and substitution until at least 70–80% is reached
- Performance of rotational thromboelastometry (ROTEM) for determination of extrinsic pathway and fibrinogen activity, with particular attention to fibrinogen substitution and adjustment of heparin dosage as required
- Aim for normothermia, and if required, initiate active warming via the ECMO heat exchange system
- Continue anticoagulation with heparin on low-dosage application

During surgery:

- Transport and positioning of the patient only in the presence of a certified perfusionist (a clinical specialist responsible for operating the ECMO circuit), if available
- Steady communication every 30 min between surgeons and anesthesiologists on updates about hemodynamic stability, blood loss, temperature, and continuous reassessment of whether and how to proceed with the surgery
- Temperature management: preheated operating rooms (at least 30 °C/86 °F), establishment of hot line system for administration of preheated fluids, active warming of body parts not involved in surgery
- Infiltration of donor areas for skin grafting with adrenalin solution or immediate soaking of areas with adrenalin gauze (solution 1:100,000). While systemic effects are not typically expected at this dilution, close communication with the perfusionist is essential during infiltration to monitor for any hemodynamic changes and ensure patient safety
- Elastic bandage of donor sites
- Stick to preoperative plan and do not continue surgery because patient seems still stable
- Stop surgery in case of hemodynamic instability or other unforeseen events such as major bleeding, or ECMO-related issues (e.g., pump alarms, oxygenator dysfunction)

After surgery:

- Transport to ICU by anesthesiologist in charge during surgery
- Maintain temperature management on ICU, preheat the room if possible
- Regular blood gas analysis (at least every 4 h for 24 h) and ROTEM and determination of factor XIII (fibrin-stabilizing factor) immediately after surgery
- Needs-oriented substitution of coagulation factors or RBCs and fluid for hemodynamic stability
- At 4 h postoperatively, assess all surgical dressings for signs of bleeding; if indicated, apply bedside coagulation using diathermy

## Discussion

In our department, we have implemented a standardized, multidisciplinary perioperative protocol specifically designed for the surgical treatment of burn patients supported by veno-venous extracorporeal membrane oxygenation (V-V ECMO). This comprehensive protocol incorporates a detailed preoperative coagulation assessment, including viscoelastic testing (e.g., ROTEM); continuous, real-time, interdisciplinary communication between surgeons, anesthetists, perfusionists, and intensive care physicians; clearly defined intraoperative decision points regarding maximal surgical duration, and structured hemodynamic and bleeding monitoring. Our experience indicates that this approach is both feasible and safe, with a low incidence of major intraoperative complications, such as bleeding or hemodynamic instability. Only in two cases was surgery terminated prematurely due to clinical concerns. Overall, the protocol significantly minimized perioperative risks and enabled timely surgical interventions, even under complex extracorporeal support conditions.

Patients with burns requiring ECMO are a particularly challenging and high-risk group due to critical respiratory failure, altered coagulation status, and the frequent need for multiple surgical procedures. Previous studies have documented bleeding complication rates of up to 50% among ECMO patients, and burn patients are particularly vulnerable due to extensive tissue injury and systemic inflammation. Coordinated interdisciplinary management is therefore essential to optimize timing, anticoagulation, and surgical approaches.

While the results are encouraging, the retrospective, single-center nature of our analysis and the limited sample size necessitate cautious interpretation. Larger, prospective, multicenter studies are essential to validate this standardized perioperative management approach, identify predictors of complications, and potentially tailor anticoagulation and surgical timing protocols to individual patient risk profiles. Integrating specialized burn-ECMO patient data into international registries would facilitate evidence-based improvements and consensus development, thereby enhancing outcomes for this vulnerable group.

## Data Availability

All data is provided within the manuscript.

## References

[CR1] Buchtele N, Levy JH. Between a rock and a hard place: anticoagulation management for ECMO. Med Klin Intensivmed Notfmed. 2024;119(Suppl 2). 10.1007/S00063-024-01116-0 10.1007/s00063-024-01116-0 PMC11579092 38457000

[CR2] Chiu YJ, Huang YC, Chen TW, King YA, Ma H. A systematic review and meta-analysis of extracorporeal membrane oxygenation in patients with burns. Plast Reconstr Surg. 2022;149(6):1181E-1190E. 10.1097/PRS.0000000000009149. 35426867 10.1097/PRS.0000000000009149 PMC9150852

[CR3] Frost S, Davies L, Porter C, Deodhar A, Agarwal R. A review of burn patients requiring extracorporeal membrane oxygenation at a burns facility. Eur Burn J. 2021;2(4):293–300. 10.3390/EBJ2040021.

[CR4] ELSO General Guidelines Extracorporeal Life Support Organization (ELSO) General guidelines for all ECLS cases. Published online 2017. Accessed July 30, 2025. www.elso.org.

[CR5] Fina D, Matteucci M, Jiritano F, et al. Extracorporeal membrane oxygenation without systemic anticoagulation: a case-series in challenging conditions. J Thorac Dis. 2020;12(5):2113. 10.21037/JTD.2020.04.54. 32642115 10.21037/jtd.2020.04.54 PMC7330289

[CR6] Hebert S, Erdogan M, Green RS, Rasmussen J. The use of extracorporeal membrane oxygenation in severely burned patients: a survey of North American burn centers. J Burn Care Res. 2022;43(2):462–7. 10.1093/JBCR/IRAB103. 34091669 10.1093/jbcr/irab103

[CR7] Jung C, Stueber T, Mirus M, Heubner L, Spieth PM. Anticoagulation in venovenous extracorporeal membrane oxygenation. Front Med (Lausanne). 2025;12: 1530411. 10.3389/FMED.2025.1530411. 40103791 10.3389/fmed.2025.1530411 PMC11913846

[CR8] Martucci G, Giani M, Schmidt M, et al. Anticoagulation and bleeding during veno-venous extracorporeal membrane oxygenation: insights from the PROTECMO study. Am J Respir Crit Care Med. 2024;209(4):417–26. 10.1164/RCCM.202305-0896OC. 37943110 10.1164/rccm.202305-0896OC

